# Supplementary figures and images for: Lymphoid-specific helicase inhibits cervical cancer cells ferroptosis by promoting Nrf2 expression
Source: PeerJ. 2023 Nov 28;11:e16451. doi: 10.7717/peerj.16451 (PMC10691353; doi:10.7717/peerj.16451)

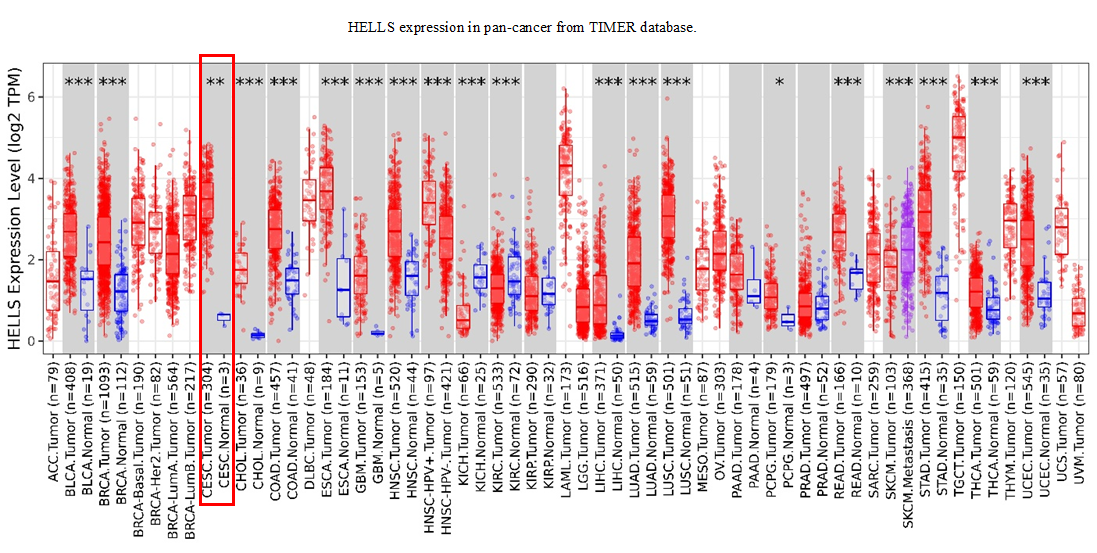

Supplement: Supplemental Information 1 [file peerj-11-16451-s001.png]

Fig2B HELLS

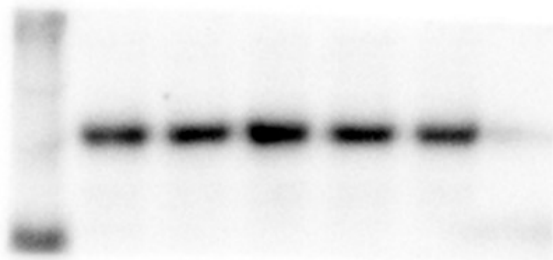

Fig2B actin

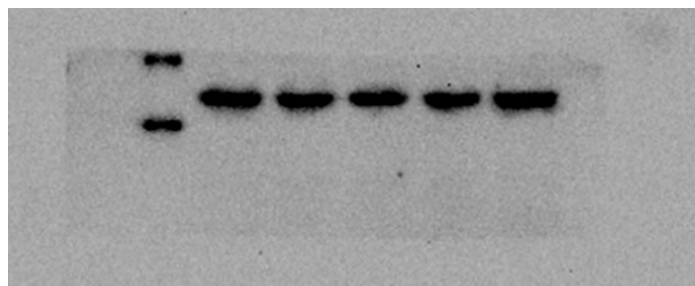

Fig2D HELLS

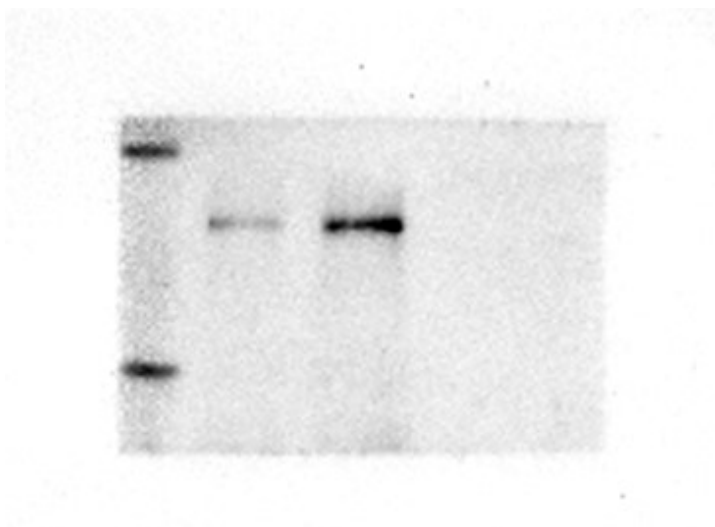

Fig2D actin

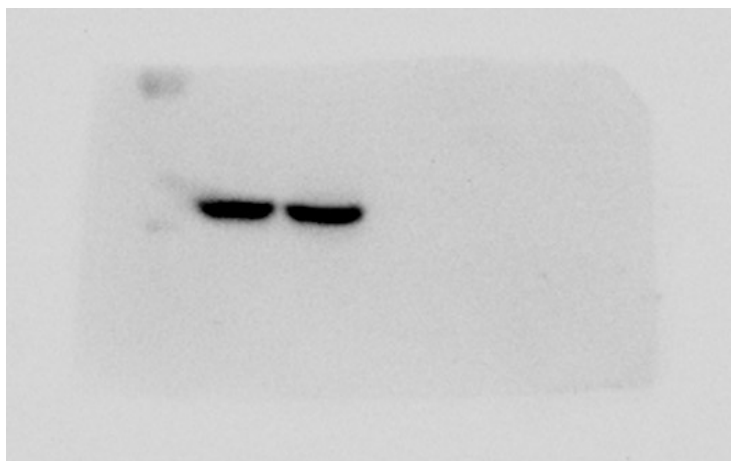

Fig3B HELLS

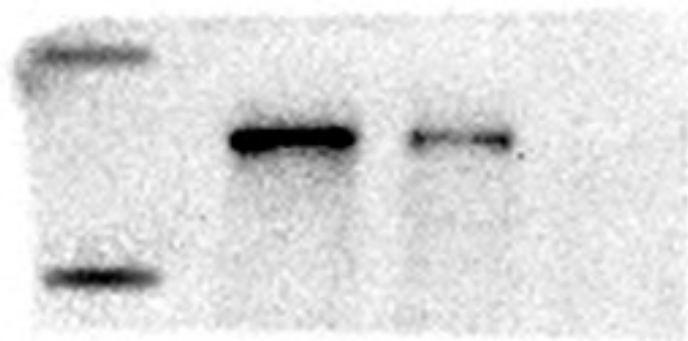

Fig3B actin

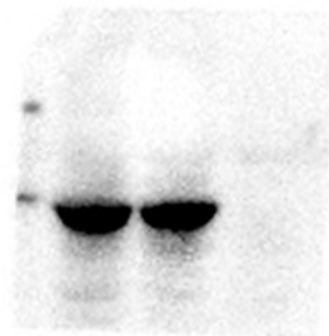

Fig5C NRF2

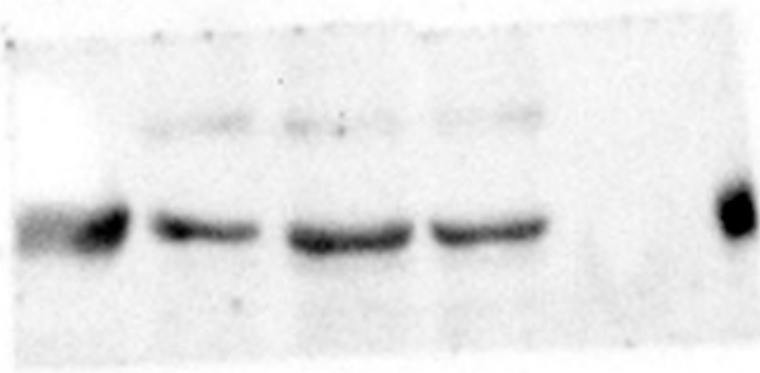

Fig5C actin

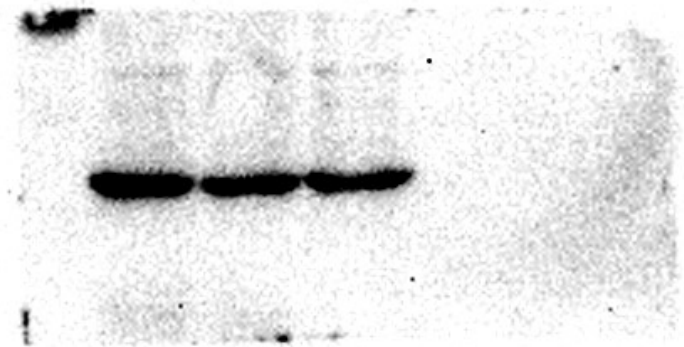

Supplement: Supplemental Information 2 [file peerj-11-16451-s002.pdf]
